# Thoracoscopic repair of diaphragmatic hernia following ventricular assist device implantation

**DOI:** 10.1186/s40792-020-00933-7

**Published:** 2020-07-11

**Authors:** Ryuta Saka, Takaaki Sakai, Tomomitsu Kanaya, Yuko Tazuke, Yosuke Kugo, Masaki Taira, Takayoshi Ueno, Hiroomi Okuyama

**Affiliations:** 1 Department of Pediatric Surgery, Osaka University Graduate School of Medicine, 2-2 Yamadaoka, Suita, Osaka, 565-0871 Japan; 2 Department of Cardiovascular Surgery, Osaka University Graduate School of Medicine, Osaka, Japan

**Keywords:** Diaphragmatic hernia, Thoracoscopy, Ventricular assist device

## Abstract

**Background:**

Diaphragmatic hernia is a rare complication of ventricular assist device (VAD), mainly developing after explantation of the device. We herein report a case of diaphragmatic hernia that developed following the implantation of VAD.

**Case presentation:**

A 4-month-old girl with a diagnosis of dilated cardiomyopathy underwent VAD implantation for a bridge to heart transplantation. Three months later, intermittent vomiting developed, and left-sided diaphragmatic hernia was confirmed on plain X-ray and computed tomography. Without any findings of ischemia, we performed elective thoracoscopic repair of the diaphragmatic hernia. In the right decubitus position, thoracoscopy revealed the small intestine to be herniated into the left thorax. After reduction of the herniated intestine, the defect of the diaphragm (3 × 2 cm in size) was directly closed with interrupted non-absorbable sutures. Her postoperative course was uneventful.

**Conclusion:**

Thoracoscopic repair of diaphragmatic hernia associated with VAD implantation may be a safe approach.

## Background

Left ventricular assist devices (LVADs) have become popular in children with the advent of the Berlin Heart EXCOR® (BHE) VAD (Berlin Heart, Inc., The Woodlands, TX, USA). The BHE VAD provides long-term circulatory support for pediatric patients with severe heart failure, not only functioning as a bridge to transplantation but also facilitating the successful weaning off of a VAD [1]. Although advances in VAD technology and clinical management have resulted in improved outcomes of pediatric heart failure, VADs are associated with potential risks of neurologic, hematologic, gastrointestinal, and immunologic complications [2].

VAD implantation requires dissection of the pericardium and diaphragm from the anterior chest wall, and diaphragmatic hernia is known to be a complication of VAD. Diaphragmatic hernia complicated with VAD is usually identified after VAD explantation with or without heart transplantation [3]. Thus far, only one case report of diaphragmatic hernia in a patient with an LVAD still implanted has been published [4].

We herein report a pediatric case of left diaphragmatic hernia following BHE LVAD implantation in which thoracoscopic repair was performed.

## Case presentation

A 4-month-old girl showing severe heart failure was referred to our hospital. Echocardiography revealed remarkable dilatation of the left ventricle, severe mitral regurgitation, and a low left ventricular ejection fraction (15%), and she was diagnosed with dilated cardiomyopathy (DCM). She received temporary LVAD implantation (ROTA flow), followed by exchange to a BHE device. At the implantation of the LVAD, the pericardium and diaphragm were dissected from the anterior chest wall using LigaSure^TM^ (Medtronic, Minneapolis, MN, USA).

**Fig. 1:**
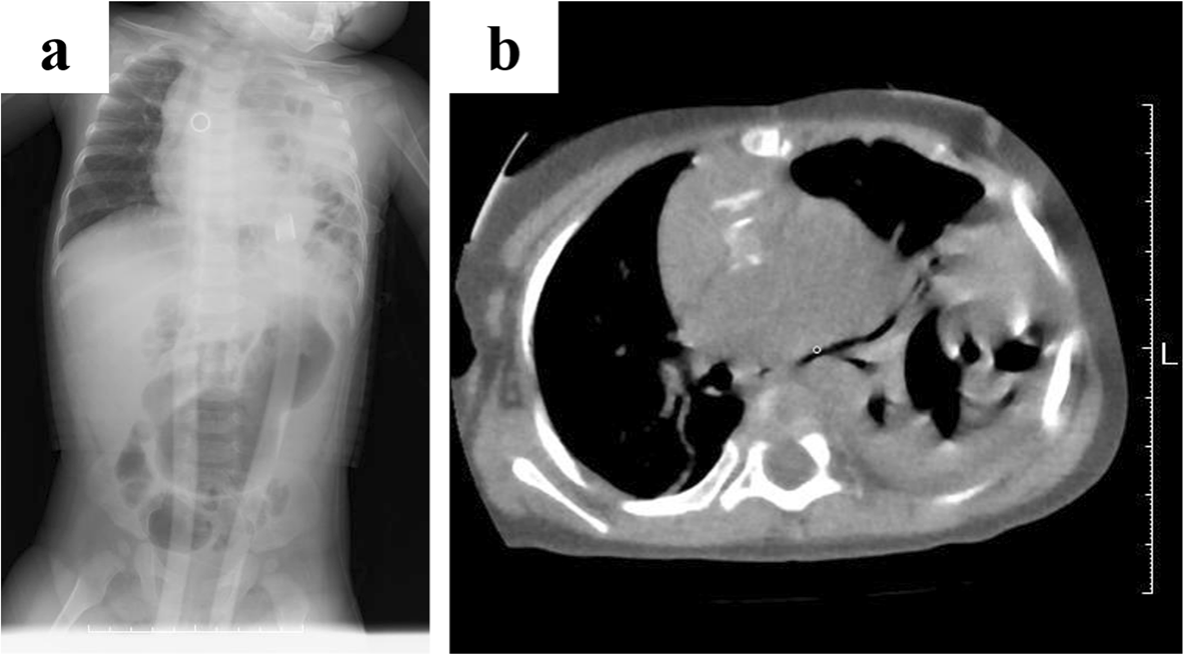
Preoperative radiological findings. **a** Plain X-ray shows suspected bowel in the left thorax. **b** Computed tomography confirmed diaphragmatic hernia

At 7 months of age (body weight, 5 kg), she received a surgical consult for intermittent emesis and irritability. Her abdomen was soft, and gurgle was heard at the left chest. Plain X-ray showed suspected intestinal loops in her left thorax (Fig. 1a). Plain chest and abdominal computed tomography confirmed the diagnosis of diaphragmatic hernia with herniation of the small intestine (Fig. 1b). The laboratory findings were normal. Ultrasonography revealed intestinal tracts with maintained blood flow in the left thorax. In the absence of findings of bowel ischemia, an operation was performed electively after decompressing the intestinal tract. Her anticoagulant management was maintained with aspirin, dipyridamole, and warfarin preoperatively. Prior to the operation, the effect of warfarin was reversed with lyophilized human prothrombin complex concentrate (Kcentra®; CSL Behring, King of Prussia, PA, USA).

**Fig. 2:**
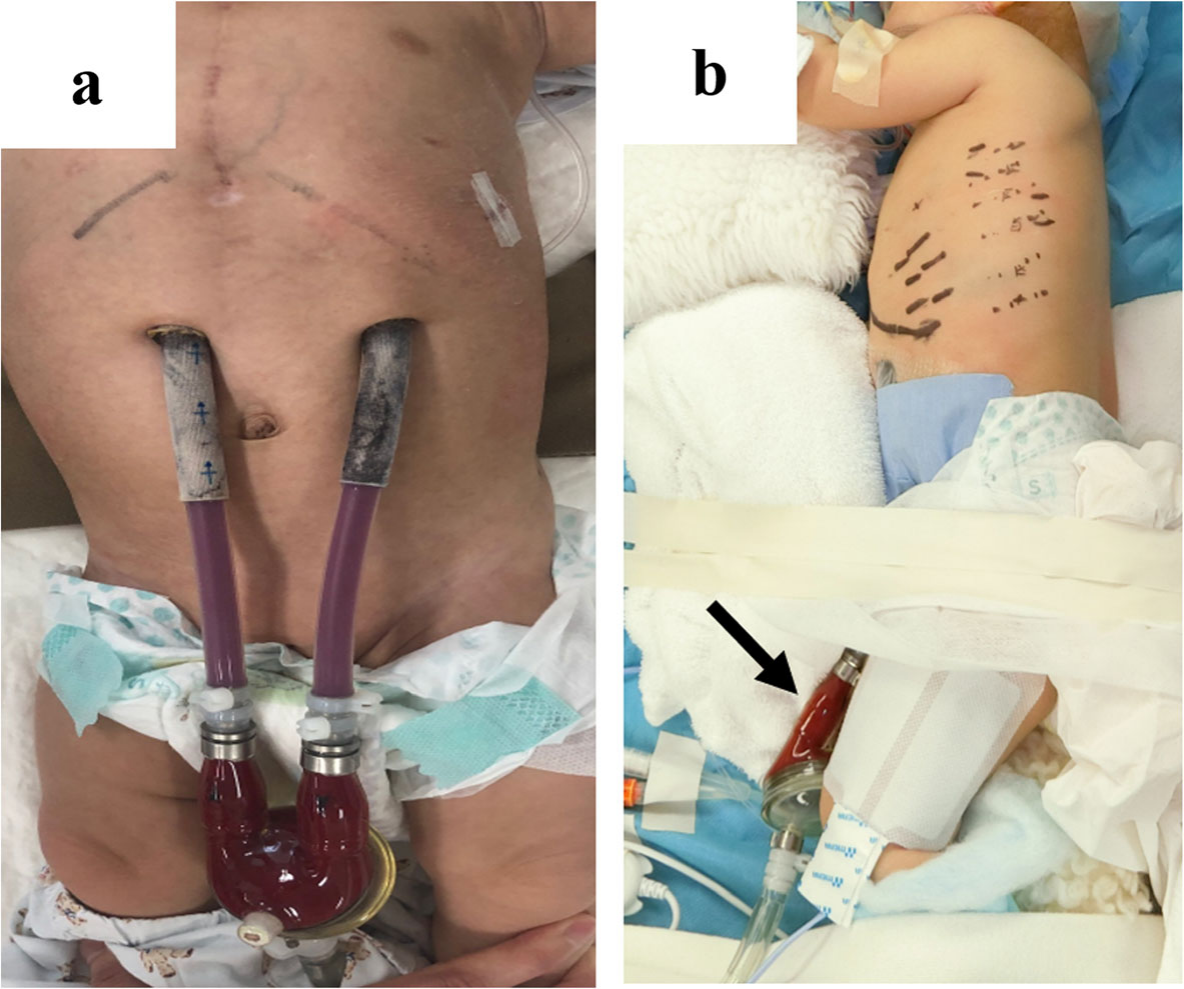
Patient’s abdomen and position. **a** VAD cannulae were inserted from the upper abdomen. **b** She was placed in the right decubitus position. The blood pump (black arrow) and cannulae were fixed to her body

**Fig. 3:**
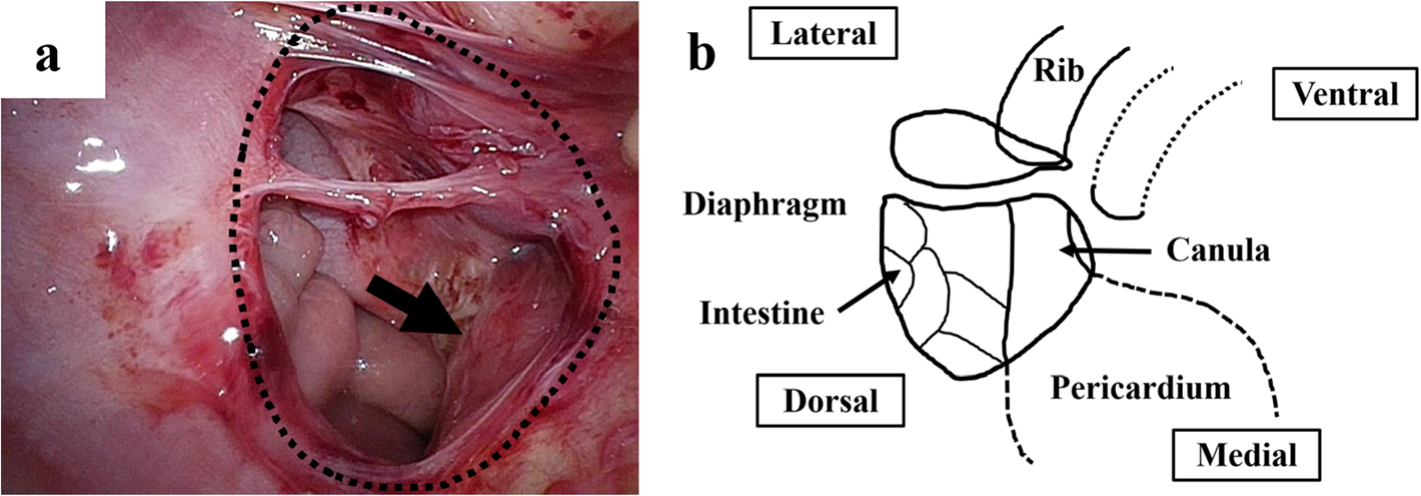
The orifice of the diaphragmatic hernia and cannula. Following reduction of the herniated small intestine, the diaphragmatic defect (dotted line) was confirmed just lateral to the VAD cannula (black arrow). The defect was 3 × 2 cm in size

**Fig. 4:**
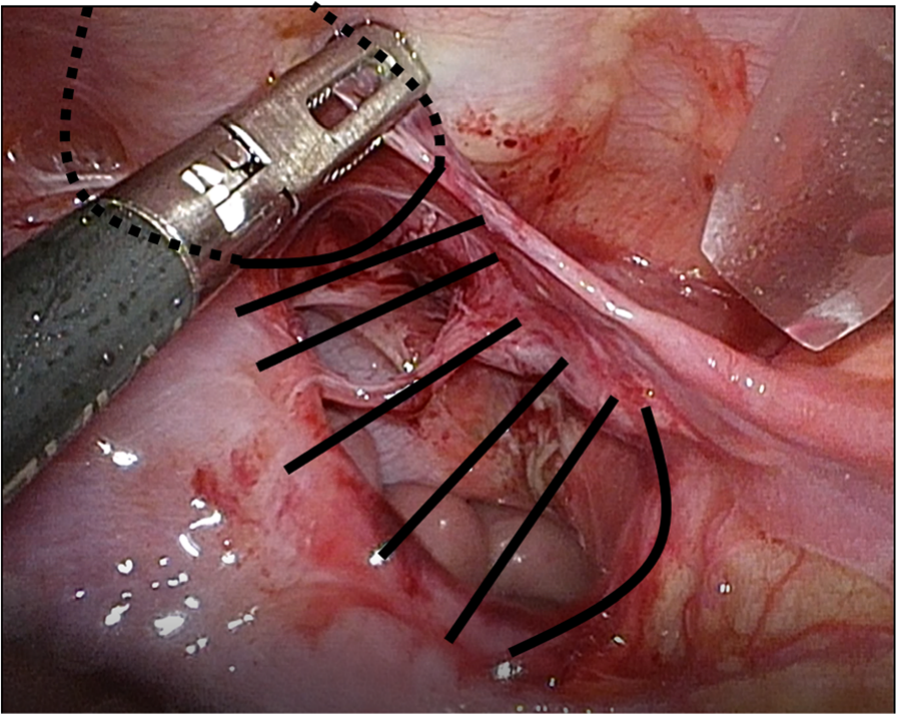
Schematic illustration of each stitch. The most medial stitch partially passed the pericardium

**Fig. 5:**
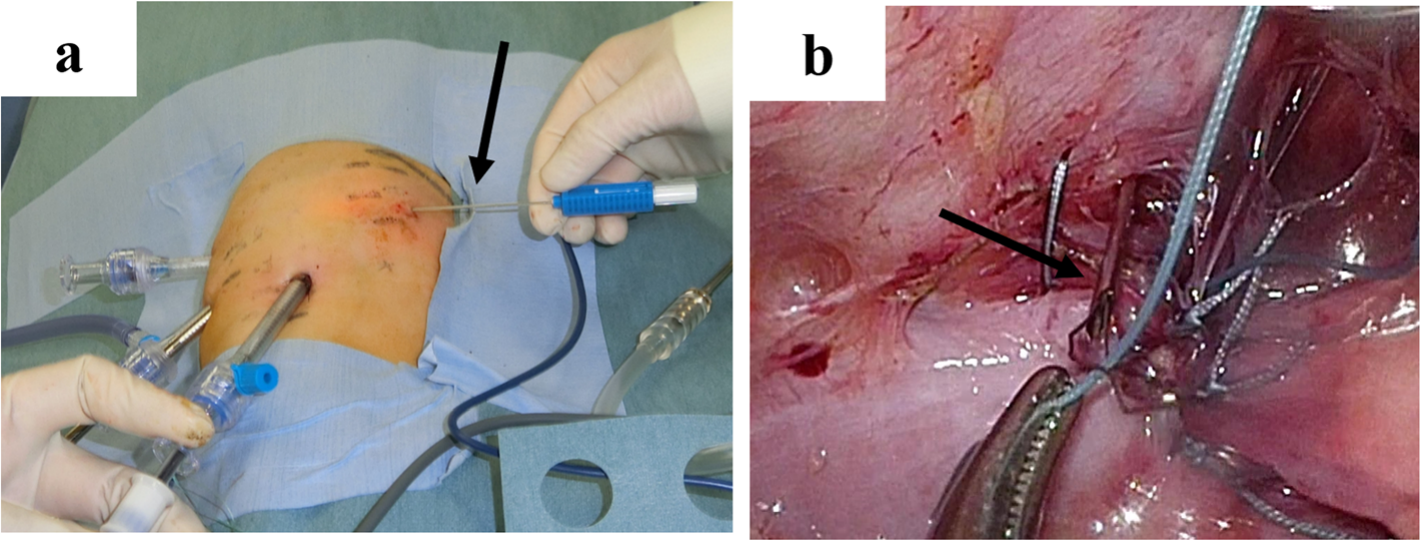
Trocar settings and ventral closure. **a** Three 5-mm trocars were inserted in the co-axial setting. Lapa-her-closure® (black arrow) was inserted at the most ventral edge of the diaphragmatic defect. **b** The ventral edge of the defect was very slight, so U-shaped sutures were achieved with the aid of Lapa-her-closure® (black arrow)

**Fig. 6:**
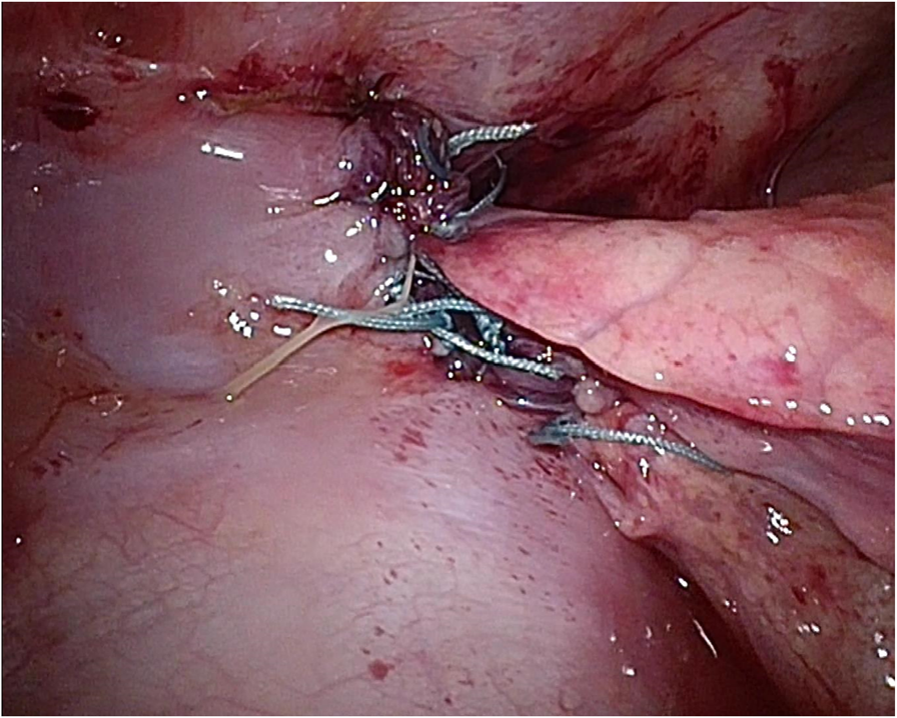
Closure of the diaphragmatic defect. Using seven non-absorbable interrupted sutures, the defect was directly closed

Since a transperitoneal approach was not possible due to the blood pump and cannulae just above the abdomen (Fig. 2a), thoracoscopic repair was performed. She was placed in the right lateral decubitus position, and BHE cannulae (inflow and outflow) were fixed to her abdomen (Fig. 2b). Three 5-mm trocars were placed at the midaxillary line at the 7th intercostal space (ICS), the posterior axillary line at the 8th ICS, and the posterior axillary line at the 10th ICS. Artificial pneumothorax (5 mmHg) using carbon dioxide was established. Following manual reduction of the herniated small intestine using a 5-mm laparoscopic blunt-tip dissector (ETHICON, Bridgewater, NJ, USA), the diaphragmatic defect (3 × 2 cm) was identified just lateral to the cannula (Fig. 3). Although the lateral rim was substantial, the medial and ventral rims were fragile. First, one thread was applied and ligated. Pulling this thread allowed us to find the rim of the orifice and peritoneum. We took care that all stitches pass through the peritoneum in both sides of the defect. The most medial thread partially passed the pericardium (Fig. 4). The most ventral knot was ligated subcutaneously with the aid of a 19-G needle (Lapa-her-closure®; Hakko, Chikuma, Japan), which has a wire loop to hold and release a thread (Fig. 5). Finally, the defect was closed with 7 interrupted sutures using 2-0 non-absorbable suture materials (Fig. 6). A 10-Fr chest tube was placed through the most caudal incision. Intraoperative bleeding was 1 ml.

Her postoperative course was uneventful, and she is waiting for heart transplantation.

## Discussion

Recently, VAD has become a well-established treatment for severe heart failure in children with the advent of the BHE VAD and the refinement of perioperative management methods [2]. BHE, which is a pneumatic paracorporeal device, is the only VAD for small patients [5] and may function as a bridge to transplantation or recovery [6]. The waiting period for heart transplantation is longer in Japan than the global average because of the severe shortage of organ donations for children, so the duration of LVAD implantation may be unavoidably long [1, 7].

Diaphragmatic hernia is a potential complication of a VAD due to the need for dissection or penetration of the diaphragm when implanting the device. Although there have been some reports of diaphragmatic hernia following LVAD explantation, most cases have been reported in patients with intracorporeal (intraperitoneal or extraperitoneal) VADs [3, 8–11]. Groth et al. reported that the risk of diaphragmatic hernia is higher after explantation of intraperitoneal LVAD (5.2%) than with extraperitoneal LVAD (0%) [8]. Ongele et al. reported the only case of diaphragmatic hernia associated with an implanted LVAD (Heartmate II) treated with robot-assisted repair [4]. This is the first report of diaphragmatic hernia in a patient with an implanted BHE device. In this case, slight damage to the left diaphragm during dissection from the anterior chest wall might lead to the diaphragmatic hernia 3 months later. Attention should be paid to diaphragmatic damage during VAD implantation.

There are two approaches to repairing diaphragmatic hernia: through the thorax or the abdomen. In our case, the patient had an extracorporeal blood pump and cannulae just above her abdomen. Therefore, we needed to select the thoracoscopic approach, which is often used in cases of congenital diaphragmatic hernia. The advantage of the thoracoscopic approach was that we could enclose the orifice without obstruction by intraperitoneal organs, such as the liver and intestine. In the present case, we were able to complete diaphragmatic hernia repair safely under an excellent thoracoscopic view. While we selected direct closure, most cases of diaphragmatic hernia following explantation of the VAD receive patch repair because the diaphragmatic defect tends to be large [4, 8–10]. Since it was acquired diaphragmatic hernia, the defect was small enough for direct closure without tension.

In our institute, aspirin, dipyridamole, and warfarin were routinely used for chronic anticoagulation management following VAD implantation. We discontinued the administration of warfarin and reversed the effect with human prothrombin complex concentrate just prior to the operation. Perioperative anticoagulation was managed with the administration of aspirin and dipyridamole with intravenous heparin infusion. Intraoperative and postoperative bleeding was slight with meticulous intraoperative hemostasis. A thoracoscopic approach requires a smaller incision than thoracotomy, resulting in little bleeding.

## Conclusions

Thoracoscopic repair can be a safe and adequate approach to managing patients with diaphragmatic hernia associated with an implanted VAD.

## Data Availability

The authors declare that all the data in this article are available within the article.

## Abbreviations

VAD: Ventricular assist device
DCM: Dilated cardiomyopathy
BHE: Berlin Heart EXCOR®
